# Making the choice between bioelectrical impedance measures for body hydration status assessment

**DOI:** 10.1038/s41598-021-87253-4

**Published:** 2021-04-08

**Authors:** Dmitry M. Davydov, Andrey Boev, Stas Gorbunov

**Affiliations:** 1 University of Jaén Hospital, FIBAO, Jaén, Spain; 2 Laboratory of Neuroimmunopathology, Institute of General Pathology and Pathophysiology, Russian Academy of Sciences, 8 Baltiyskaia ul., Moscow, 125315 Russia; 3 Aura Devices Inc., 1013 Centre Road, Suite 403-B, Wilmington, DE 19805 USA

**Keywords:** Analytical biochemistry, Biophysical methods, Experimental organisms, Computational models, Biomarkers

## Abstract

Situational or persistent body fluid deficit (i.e., de- or hypo-hydration) is considered a significant health risk factor. Bioimpedance analysis (BIA) has been suggested as an alternative to less reliable subjective and biochemical indicators of hydration status. The present study aimed to compare various BIA models in the prediction of direct measures of body compartments associated with hydration/osmolality. Fish (n = 20) was selected as a biological model for physicochemically measuring proximate body compartments associated with hydration such as water, dissolved proteins, and non-osseous minerals as the references or criterion points. Whole-body and segmental/local impedance measures were used to investigate a pool of BIA models, which were compared by Akaike Information Criterion in their ability to accurately predict the body components. Statistical models showed that ‘volumetric-based’ BIA measures obtained in parallel, such as *distance*^*2*^*/R*_*p*_, could be the best approach in predicting percent of body moisture, proteins, and minerals in the whole-body schema. However, serially-obtained BIA measures, such as the ratio of the reactance to resistance and the resistance adjusted for distance between electrodes, were the best fitting in predicting the compartments in the segmental schema. Validity of these results should be confirmed on humans before implementation in practice.

## Introduction

Frequent or long-lasting de- or hypo-hydration (i.e., body fluid deficit) has long been considered a significant health problem, because it has been found to increase morbidity, mortality, and impaired performance in the general population, specific groups such as athletes and soldiers, and patients with various diagnoses^1–3^. Water, dissolved or soluble proteins forming colloids, non-osseous minerals such as potassium, magnesium, calcium, and sodium in the form of electrolytes, are essential parts of body fluids, and their extracellular concentrations are important indicators of the body’s hydration status^4^. During fluid loss or dehydration (e.g., due to exercise), an adequate plasma volume is maintained as a homeostatic endpoint by baro- and osmo-regulatory mechanisms^5^. This is primarily determined by the overall water balance and transcapillary fluid shift, which depends on the hydrostatic and systemic blood pressures as well as the osmotic and oncotic (colloid osmotic) pressure gradient at the capillary wall^6–8^. Despite this process, dehydration is considered to be mainly related to extracellular water, while intracellular water has been proposed to minimally contribute to fluid loss, and its balance was found to be preserved in healthy subjects^7^.

The dehydration state can be diagnosed by the presence of appropriate physical signs and results of blood or other fluids biochemical tests^9,10^. One study showed that most physical signs, such as tachycardia, dry mucous membrane, dry axilla, poor skin turgor, sunken eyes, and long capillary refill time, showed poor sensitivity for detecting either (water-loss or water-and-solute-loss) form of dehydration^11^. Tests of fluids are considered to be more sensitive. For example, when water in body fluids decreases the concentrations of electrolytes (non-osseous minerals) and dissolved proteins increase, this indicates body dehydration^10^. The opposite process indicates body rehydration. However, a change in protein concentration of biological fluid samples in a study of healthy men was found to be a more reliable indicator of change in body hydration status compared with a change in electrolytes or in fluid capacity (e.g., saliva flow rate)^10^. It is proposed that the lower reliability of water and electrolyte compartments, when compared with proteins, in predicting hydration status is related to individual differences in the representation of the state of euhydration by these indicators. In other words, compared to concentration of proteins, the same value of water and electrolytes compartments of the body may represent either hypo-, eu-, or hyper-hydration status in different individuals. Thus, to be of use as potential markers of the whole-body hydration status, the water and electrolytes compartments should be adjusted for an undefined individual trait, such as body weight or height, that also affects hydration status variations. Therefore, further studies are needed of more reliable methods incorporating these considerations for detecting dehydration at home and in nursing homes.

Bioimpedance analysis (BIA) has been suggested as an alternative to the subjective and biochemical indicators of hydration status^12^. Electrical impedance-based methods have traditionally been used to evaluate both hydration (total body water amount and its compartments) and nutrition (lipid and non-lipid contents) status in both human and animal studies^13–16^, including fish^17–23^. The present study’s goal was restricted to assessing BIA models with respect to hydration status only. BIA can provide estimates of body compartments by measuring the resistance and reactance of biological tissue at various low- and high-frequency electrical currents. Resistance indicates the conductive characteristics of bodies and fluids and increases as the proportions of fat and bone content in tissue increase but decreases as the proportions of water and muscle mass content in tissue increase. Reactance is capacitor’s opposition to alternating current and indicates cell membrane capacitance, which is related to intracellular water volume. These two electrical values are used in calculations involving common electrical property equations to generate data for regression models with proximate composition measures. Many methods are available for assessing the proximate body composition of a variety of organisms as references or criterion points. A physicochemical compositional analysis is considered to be the most reliable approach for this type of assessment, but due to its destructiveness, is only permitted in animal models^22,24,25^. This direct lethal method bypasses any theoretical model of body compartment calculation restricted by non-lethal or in vivo reference methods to 2-, 3-, and 4-component models (e.g., by using hydrometry [with deuterium or tritium dilution] for body water, hydrodensitometry [with underwater weighing] for body density and then body fat, dual-energy X-ray absorptiometry [DEXA] for body bone mineral content). Most non-lethal reference methods have shortcomings, including low precision of the related body 2-, 3-, and 4-compartment models. In the present study, the fish species Cyprinus carpio was used as a model for comparison of different BIA measures and equations to predict between-subject variance in proximate body measures of hydration status (i.e., percent of total body moisture, proteins, and minerals) obtained by physicochemical analyses after destruction of the fish body.

There are various empirical and theoretical approaches for in vivo analysis of body water compartment using resistance (*R*) and reactance (*Xc*), and related phase angle (*ϕ*) derived from bioimpedance (*Z*) or conductance (*Y*) modules measured at 50 kHz. For example, an empirical relationship was established between the impedance index (*distance*^2^/*R*) and the total volume of body water (TBW), which contains electrolytes that conduct the electrical current through the body^12^. The related ‘volumetric-based’ models of whole-body BIA are built on an assumption that the resistance at 50 kHz is proportional to TBW. However, at this frequency, this empirical formula should express a weighted sum of extra-cellular (ECW; containing more chloride) and intra-cellular (ICW; containing more potassium) water resistivities. Using this methodology, individual prediction of TBW, ECW, and ICW (ICW as a difference between TBW and ECW), estimated by reference methods using deuterium or tritium dilution and sodium bromide dilution, relies on various regression models in which the formula is adjusted by population-derived indices (intercepts and slopes) to reduce inter-individual errors^12^. This study did not include the evaluation of multifrequency approaches such as bioimpedance spectroscopy (BIS). BIS was proposed as a special method for measuring indirectly ECW and TBW volumes using an assumption of parallel resistances in equivalent electrical circuit of body tissues and a specific conductivity theory to account for the presence of non-conducting elements. In BIS models, ICW can be obtained as a difference between TBW and ECW. However, most assumptions of the BIS models require their own separate study. For example, the validity of most BIS equations for assessing ICW effects on impedance measuring, as well as the validity of the parallel resistance model for BIS are questionable or not well justified using biophysical principles^26–29^.

A further review of various body hydration assessment methodologies proposed that the accuracy of traditional whole-body BIA methods in the prediction of more direct methods of body water assessment, such as dilution, was affected by the inhomogeneous nature of various body compartments and a large inter-individual variation in differences of circumferences between various body segments, for example, between the thigh and trunk^30^. Moreover, the whole-body BIA method may be limited because a larger variation in total body volume may result in relatively smaller variations in body resistance and reactance that are below the BIA precision level^30^. Furthermore, hypo- and hyper-hydration studies suggest that electrolyte balance influences whole-body BIA measurements independent of fluid changes^12,31^. All of these factors limit the applicability of predictive equations generated by whole-body BIA models. These errors limit the clinical usefulness of the current BIA methods for the assessment of body hydration in individual patients. Thus, investigation is needed of other models that may be more valid and can guarantee BIA results uncorrupted by these inter-individual and measurement errors. A segmental approach in BIA has been developed to decrease BIA errors related to inter-individual variability in body shape and difference between cross-sectional areas^12,32^. Bioimpedance of body segments is considered to behave as if the segments are in series with each other, with shorter and thicker segments contributing less to the total resistance.

Bioimpedance measures can be obtained by in series or in parallel electrical compensation schemes. Some researchers propose that the physiology of the body is best represented by the equivalent electrical circuits when ECW and ICW reside adjacent to each other in a parallel arrangement, with the ICW being isolated from the ECW by a nonconducting membrane similar to the insulating material within a capacitor^16^. Other models suggest that the ECW and ICW pathways are composed of a resistor and a capacitor in series, but do not run side by side, and thus the resistance and reactance are additive^16^. Thus, a valid model must guarantee that ECW differences do not corrupt the ICW and vice versa.

In addition, bioelectrical impedance vector analysis (BIVA) was proposed as an alternative method that does not rely on group-derived indices (i.e., decreasing the errors related to population-derived BIA models) to predict individual hydration status^12,33^. BIVA measures resistance and reactance, and standardizes them for distance between electrodes using length (*L*) or height (*H*) for different electrode locations in order to assess the variability in hydration status of subjects^12^. These variables can also be used separately to predict individual extra- (*ECWi* = *R/L*) and intra- (*ICWi* = *Xc/L*) cellular water, respectively, or as an impedance vector length (calculated as the hypotenuses of individual impedance values adjusted to distance, either *L* or *H*) to predict individual total body water TBWi = $\sqrt[2]{{ICWi^{2} + ECWi^{2} }}$. For example, in response to exercise, an increase in *Xc*/*L* (i.e., an increase in intracellular fluid) was significantly related to greater osmolarity or more dehydration, which indicated a probable compensatory fluid shift from the ECWi to the ICWi compartment during dehydration extending the length of the bioimpedance vector^14^. However, this study did not include BIVA models with resistivity measures (specific resistance and reactance, Ω m) based on an assumption that body impedance is affected by cross-sectional area, besides the conductor length, frequently represented by height. The comparison of the specific (resistivity corrected) BIVA measures with classical (uncorrected) BIVA measures showed that former measures were more accurate in predicting only measures of nutrition status (e.g., relative fat mass, %FM), but the later measures were more accurate in predicting mainly measures of hydration status that were the main target of the present study.

However, most BIA techniques require further evaluation, because in most cases, the consistency and accuracy have been assessed against references or criterion techniques, such as the dilution methods, which have their own shortcomings (e.g., low precision of their measurement, deuterium loss in urine and breath, and some differences of intracellular and transcellular penetrations between genders and in individuals with different homeostasis)^16,34^. Besides these indirect techniques, BIA models can also been developed using animals that can be terminated, such as fish, to allow for direct measurements of moisture and other compartments of the body associated with hydration/osmolality as proximate composition references or criterion points^22^. Such direct or proximate composition references obtained using lethal physicochemical methods should show more reliable associations with BIA measures even without populationally-derived regression models that are biased by various theoretical viewpoints on body compartments. Moreover, relationships of proximate composition measures obtained in the animal models by these destructive methods to BIA data are considered to be similar to those of humans^20^. However, formulas for predicting absolute body compartment values will require adjustment to regression constants that should differ between animal (e.g., fish) and human populations, but this was not an objective of the present study.

Thus, this research was conducted on an animal (fish) model using destructive physicochemical methods to obtain criterion points for hydration measures (water, proteins, and minerals) together with fat as a component of nutrition status to compare various BIA modelling and measurement techniques and thus to determine the best practices for the use of BIA data. The following hypotheses were proposed and tested in the current study: (i) resistance (*R*), reactance (*Xc*), and total bioimpedance (*Z*), and related indices derived from standardized and non-standardized equations would significantly predict body moisture/water, proteins, and ash/minerals each obtained by proximate composition analysis as different reference points of hydration status; (ii) parallel and serial electrical compensation schemes for calculating *R*, *Xc*, and *Z*, and related indices would predict the hydration status reference points with different power associated with their different relative distribution between ICW and ECW in the individual; (iii) compared with a whole-body BIA approach, a segmental approach in BIA would show best fitting results for predicting proximate body compartments associated with hydration status with lower dependence on inter-individual variability in body weight and/or length; (iv) a better hydration status would be associated with decreasing *R* and increasing *Xc* (indicating parallel increases of ECW and ICW), increasing in both* R* and *Xc* with a higher *Xc* to *R* ratio (indicating transfer of ECW to ICW).

## Materials and methods

### Fish, experimental conditions, and design

The Royal or Mirror Carp species (*Cyprinus specularis* or *C. rex. cyprinorum* L.) was selected as a biological model in this study, because most individuals of this species lack scales, and their skin may be naked even at long intervals^35^. These scale-free locations on the fish's body were where non-invasive contact electrodes were placed while invasive needle electrodes were placed at locations where scales existed. Living subjects for the study were obtained from a local population of Mirror Carp in a fish farm of Belgorod Region, Russia.

**Figure 1:**
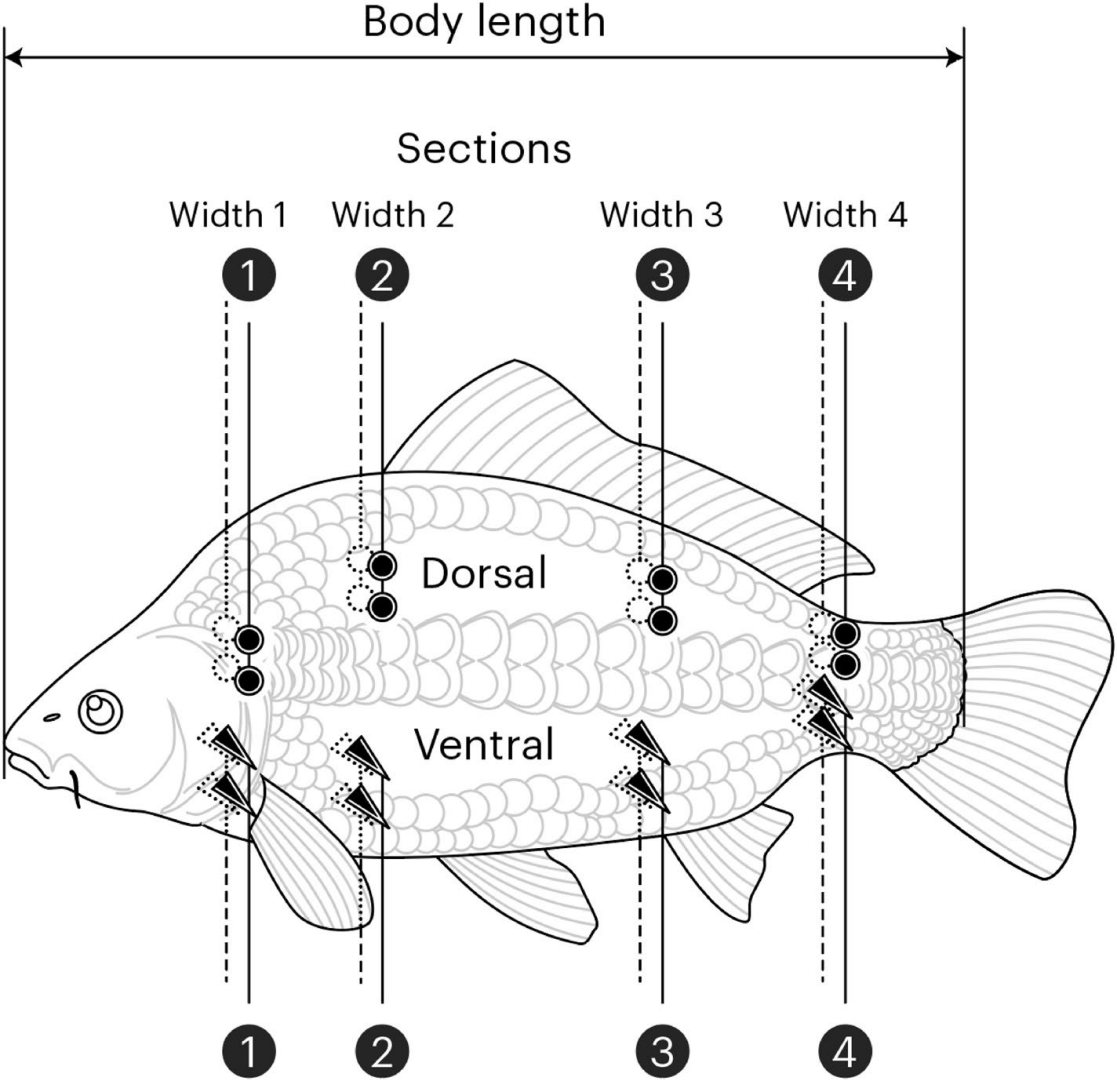
Derivations from four dorsal (by contact gold electrodes, 5 × 10 mm) and four ventral (by stainless steel subdermal needle electrodes, d = 0.35 mm and length = 10 mm) points of two-side segments of the body of fish at dorsal and ventral rows. Space on vertical line between paired on one side electrodes, including signal-emitting (higher) and signal-detecting (lower) electrodes, was kept constant (3 mm between closest edges for contact and 7.5 mm for needle electrodes). Distances between each paired sets of electrodes (i.e., width between sides of the segments) were individually varied for every segment within and between subjects (see Table 1).

The total sample for the study included 20 mature carp, with five studied per day. Each selected carp was initially immobilized by cranial concussion^36^ then measured to the nearest millimetre for standard length (*L*; from the tip of the nose to the end of the caudal peduncle) and width (*W*; at 4 body locations; see Fig. 1), weighed to the nearest gram (*Wt*), and assessed by two different BIA procedures (BIA of 4 segments with contact and needle electrodes and BIA of the whole body with contact and needle electrodes). The potential effects of time on BIA after death were considered to be minimized in this design^37^. Subsequently, carps were individually labelled and wrapped in aluminium foil to reduce moisture loss, bagged with constant t = 2–5 °C, and immediately sent for same-day proximate analysis of composition (less than 6 h after fish were sacrificed) at the CCI certified laboratory in the Voronezh Region, Russia. All working procedures complied with the European Union Directive 2010/63/EU for the protection and welfare of animals used for scientific purposes, with the ARRIVE guidelines, and were approved by the Animal Ethics Board of the Institute of General Pathology and Pathophysiology, Russian Academy of Sciences.

### BIA readings

For BIA procedures, fish were blot dried and placed on a nonconductive board. After attaching electrodes, parallel conductance module (*Y*) and serial phase angle (*ϕ*) readings for each fish were immediately obtained with 0.15% of accuracy by applying 50 kHz sinusoidal current with 40 mV using an immittance (RLC) analyser E7-25 (MNIPI, Belarus) with a valid calibration certificate. The segmental readings were acquired from four dorsal (by contact gold-plated electrodes, 5 × 10 mm) and four ventral (by stainless steel subdermal needle electrodes, d = 0.35 mm and length = 10 mm) segments across each side (left and right) of the body of each fish (Fig. 1). The needle electrodes were positioned to penetrate approximately 5 mm into each fish. Whole-body readings were acquired from two dorsal (anterior and posterior) locations by the same contact electrodes and two ventral (anterior and posterior) locations by the same needle electrodes on the left side of each fish. Dorsal electrode locations were proposed to be more related to moisture BIA models, while ventral electrode locations were proposed to be more related to lipid BIA models^37^. In the present study, readings from dorsal and ventral locations were statistically linked in analyses to obtain integrated BIA measurement effects (i.e., two-surface statistical BIA models) in predicting body components by both ‘segmental’ and ‘whole-body’ BIA schemas. The two-surface models were proposed to incorporate greater amounts of information about the internal composition of a subject, which could improve their predictive ability. Previously, the combination of dorsal and ventral BIA data in two-surface BIA models was found to be a more precise estimator of the percent of proximate moisture content of the body than the models developed from either dorsal or ventral data alone (i.e., from single-surface BIA models)^37,38^. To minimize potential errors in BIA measurements of fish, each experiment was controlled for correct electrode locations, procedure deviations, time after death, and temperature following prior study recommendations^19^. For the whole body, a multifrequency BIA (50, 200, and 500 kHz) was acquired, but only 50 kHz was used in the present study to match with the segmental BIA obtained only by 50 kHz current.

Thus, for segmental derivations, 8 pairs of electrodes (i.e., 4 contact and 4 subdermal) were placed opposite each other on different sides of the body. While space on the vertical line between paired (i.e., signal-emitting [higher] and signal-detecting [lower]) one-side electrodes was kept constant (3 mm between closest edges or 13 mm between electrode centres for contact and 7.5 mm for needle electrodes), the distances between each paired sets of between-side electrodes individually varied for every segment within and between subjects depending on widths of the segments (see Table 1 for means and SDs for all dorsal and ventral segments’ readings). Thus, the distances between pairs of electrodes of segmental BIA were dependent on the width of the body on the respective segments/sections from the anterior through the middle to the posterior, while the distances between pairs of electrodes of the whole-body BIA were dependent on the length of the body (Fig. 1).

**Table 1:** Body weight, body length, width at different (1–4) sections, condition factor (K), and compositions (%) of individuals (N = 20).

Variables	Mean	Ranges of values	
		Min	Max
Body weight (kg)	1.149	0.702	1.565
Body length (mm)	340	270	390
Width1 (mm)	63	44	74
Width2 (mm)	57	41	70
Width3 (mm)	30	22	48
Width4 (mm)	15	11	20
Moisture (%)	76.8	67.3	82.7
Protein (%)	17.18	14.44	18.45
Lipid (%)	5.4	1.4	13.8
Ash (%)	1.08	0.74	1.55
K (g/cm^3^)	2.94	2.23	3.57

For each segmental and whole-body BIA assessments, parallel conductance module (*Y,* siemens) and serial phase angle (*ϕ,* degrees) measures were collected three times and further expressed as averages. Room temperature was controlled during all BIA procedures to ensure that the examined fish had similar body temperatures. During BIA procedures, the mean (SD) room temperature was 23.2 ± 2.5 °C. The conductance module and phase angle readings were further used as input for different bioelectrical equations to determine resistance and reactance in series (*R,* Ω and *Xc,* Ω) and in parallel (*R*_*p*_, Ω and *Xc*_*p*_, Ω), as well as other unstandardized and standardized indicators, such as impedance (*Z* = (*R*^2^ + *Xc*^2^)^0.5^, Ω), that were expected to be related to hydration balance (see all used equations in Supplementary Table S1).

This study did not include BIA models with resistivity measures (specific resistance and reactance, Ω m) of whole body and each body segment based on the simple assumption inherent to Ohm’s law that body impedance is affected by cross-sectional area, besides the conductor length, frequently represented by height in humans. The main goal of the study was to compare and to validate BIA measures and equations for predicting fluctuations in the most important components of hydration status (hypo-, eu- or over-hydration) and hydration level (de- and re-hydration) such as relative content of water, proteins, and minerals but not for computing absolute (in kg) or relative (in %) means of body fat mass and body fat free mass from BIA measures as main indicators of nutrition status, as in studies examining nutrition and metabolic disorders^39,40^. The comparison of classical uncorrected BIVA measures with resistivity corrected BIVA measures showed that the latter measures were more accurate in predicting only measures of nutrition status (e.g., relative fat mass, %FM), but the former measures along with phase angle (Xc/R; unaffected by the choice between the classic and specific models) were more accurate in predicting mainly measures of hydration status (total body water, ECW/ICW ratio, and ICW)^41^.

### Proximate body composition analysis

Physicochemical analysis of cadavers is considered the most accurate approach to measuring human body composition to obtain reference or criterion points^42^. In the present study, after each fish was euthanized by cranial concussion and following BIA, whole-body physicochemical composition analysis of individual fish was conducted on the same day using the same laboratory procedures that were adopted for dietary analyses^43^, including a separate direct destructive determination of total body water/moisture, crude protein (as nitrogen; protein was considered to be distributed, dissolved and diluted, in ECW and ICW compartments presented in soft tissues together with undissolved forms and did not correlated with the latter), crude fat/lipid (by solvent extraction), and minerals (as ash; in this case, non-osseous minerals were considered to be distributed as electrolytes in ECW and ICW compartments presented in soft tissues and did not correlated with bone mineral mass), and were run in triplicate, averaged, and expressed as a percentage of the weight of the sample.

The main reason for assessing prediction effects of BIA measures and equations on relative but not absolute estimates of hydration status was high correlation of absolute value of total body water with body weight (34–68% of explained variance) and much lower correlation with a factor affecting homeostatic difference in water balance, e.g., associated with age (about 10% together with stature)^44^. Age is considered one of main factors affecting hydration status in humans^45^. A healthy hydration status was also associated with biological (osmo- and baro-reflex) mechanisms sensing and maintaining homeostatic (i.e., relative) water-electrolyte and osmotic equilibrium or balance but not with absolute values of hydration compartments in various body fluids^5^. In addition, absolute measures of hydration status were not recommended for the evaluation of water distribution between the extra- and intra-cellular spaces^41^.

In the present study, fat percentage from the proximate body composition analysis was considered as a reference criterion point such that best fitting BIA models for hydration were independent of this probable confounding factor as a main source of metabolic water during fasting in some animals, and thus it functioned as an additional control for well-being of the sample^46^. Fat percentage was found to be weakly associated with uncorrected bioimpedance measures^41^.

Therefore, best fitting models in prediction of variations of relative body compartments, such as water, proteins and minerals associated with differences in hydration status and fluctuations in hydration level, but not the prediction of variations of absolute or relative means of body compartments, such as fat mass and fat free mass, was the main target of the present study.

Another reason for the choice of relative proximate values in the present study was related to the consideration that since most BIA models included the total length of the body in their equations, their ability to accurately predict the absolute body components closely connected to body weight could simply be related to the adjustment of BIA measures for body length or length squared^17^.

### The fish condition analysis

The fish condition or ‘well-being’ was calculated using the expression *K* = (*Wt*/*L*^3^) × 100, where *K* = Fulton’s condition factor, *Wt* = total weight, and *L* = total length^47^. BIA models that did not include weight or length as components were evaluated for relationships to this ‘well-being’ measure to exclude biases imposed by these morphological variables^17^.

### Statistical analyses

Generalized Linear Mixed Models (GLMM) were used to derive predictive relationships of bioimpedance and morphological measures with proximate measures of body compartments. Independent variables or predictors were ‘body mass’ (*Wt*) and ‘inter-electrode distance’ (Width, *W* or Length, *L*) alone in simple models, the BIA-derived measures such as impedance index (*distance*^*2*^/*R*) in ‘conductor volume’ models (i.e., volumetric-based calculations of electrical variables), and single (*R* or *Xc*) and combine (e.g., *R* and *Xc*, *R *× *Xc*, or *Xc*/*R*) BIA measures in various non-volumetric BIA models with and without adjustment to distances between electrodes through mathematical equations (e.g., *R*/*L*) or through their respective regression coefficients (e.g., *R* to *L*). Dependent variables were direct or proximate measures of body compartments such as water, proteins, ash/minerals, and fat obtained by physical and chemical methods and adjusted to individual weight (i.e., in percent).

Whole-body BIA models with two surface one-side derivation schema (i.e., when the current was applied between pairs of 1–4 dorsal and between pairs of 1–4 ventral electrodes located at one side) and local or segmental BIA models of derivation schema with four segmental two-side pairs of electrodes (i.e., when the current was applied cross body at the anterior, two intermediate, and the posterior sections) nested into two-surface derivation schema (two-side segments at dorsal and ventral rows) (Fig. 1) were compared independently for predictive ability of percent of body water, protein, ash/minerals, and fat/lipid compartments by treating 2 surface derivations (whole-body BIA models) or 2 surface and 4 segmental derivations (local or segmental BIA models) as 2 or 2 × 4 repeated measurements in the same GLMMs. Complete independence of BIA measures was assumed across subject blocks with each BIA derivation. Thus, 40 and 160 independent BIA samples were expected to predict proximate body compartments in whole-body and segmental/local BIA models, respectively. According to the Akaike Information Criterion (AIC), diagonal covariance type was the best fitting structure for the repeated measurements. Robust method was used for computing the parameter estimates covariance matrix to protect against a probable violation of the model assumptions.

A previous study proposed that segmental bioimpedance indices can be used not only for predicting segment composition but also for estimates of whole body composition from the sum of segmental composition estimates^48^. Moreover, selected demarcation that exclude materially representative amounts of tissue with extra-cellular (ECW) and intra-cellular (ICW) water contents could not be provided for any of the individual localized body segments in order to represent the whole-body hydration level accounting for water and electrolytes. This is especially relevant for transferring findings from such animals as fish to humans having numerous differences in their anatomical constructions. Thus, in the present study, the terms segmental and local were used interchangeably to represent body places or body portions, but not the whole-body, in predicting components affecting hydration level of the total fish body. This contrasts with similar terms usually applicable to the human body to differentiate anatomical body portions (i.e., arms, trunk, and legs) as segmental derivations for BIA from local derivations for BIA of specific muscles or muscles portions.

In testing the study hypotheses, the difficulty was not related to obtaining significant correlations of bioelectrical impedance to hydration status measures but rather to specifying its different relationship to different hydration-related compartments (water, dissolved proteins, electrolytes) because of an expected high degree of intercorrelation between them in healthy individuals. Thus, any impedance parameter that was found to be correlated with one of the hydration-related compartments (e.g. water) would be expected to correlate almost equally well with the other compartments affecting hydration, such as dissolved proteins and non-osseous minerals (i.e., electrolytes), without necessarily being a specific measure of those compartments. A second-order AIC with a correction for small sample sizes was used to rank significant models of the relation of BIA data to proximate composition for selecting the most parsimonious ones (i.e., the best fitting models with the minimal AIC)^49^. SPSS 21 was used to perform all statistical analyses and to evaluate effects at α = 0.05 level of significance with t-statistic, p-value and 95% confidence interval of the prediction (95% CI) showing, respectively, the likelihood that the effect found was different from zero, the probability that the effect could purely be assigned to chance, and precision/size of the estimated effect.

## Results

### Segmental BIA relations to compartments in percent obtained by proximate composition analysis

#### Percent of total body moisture

Significant negative effects on total body water or moisture percentage assessed by proximate composition analysis were found for both in series and in parallel obtained segmental BIA measures: resistance and reactance with and without adjustment to widths of segments, the reactance-to-resistance ratio, and various total impedance measures calculated by different equations (Supplementary Table S2). According to AIC, addition of body weight as a covariate did not improve the predictive ability of the models, but prediction of total body moisture percentage was improved if the segmental BIA models also included body length (Supplementary Table S3). According to AIC, the best fitting models were a segmental BIA model of a serially obtained reactance with the effect adjusted for body length and a model of a more complex equation involving the product of serially obtained reactance and resistance adjusted for total impedance with the final effect adjusted for body length. The best fitting models for water-related effects from Supplementary Tables S2 and S3 were presented in Table 2. Thus, the quantity of water assessed segmentally or locally by reactance or a more complex bioimpedance-related equation could better represent the percent of body moisture or body hydration status after accounting for its distribution along the whole length of a particular body.

**Table 2:** Best fitted effects of segmental bioimpedance measures on body water/moisture, proteins, and ash/minerals (in percent).

Measures	Moisture (%)						
Name	Schema	Obtained	Symbol	Equation	t(p)	95% CI	AIC
**Model 1**							
Reactance, Ω	Serial	Calculated	*Xc*	(1/*Y*) × *sin*(*ϕ*)	− 3.312 (0.001)	− 3.282 to− 0.830	857.459
**Model 2**							
Intra-cellular water	Serial	Calculated	ICW*s2*	*Xc/R*	− 2.088 (0.038)	− 9.918 to − 0.276	854.478
**Model 3**							
Reactance, Ω	Serial	Calculated	*Xc*	(1*/Y*) ×* sin*(*ϕ*)	− 4.159 (5.200E−5)	− 2.583 to − 0.919	**844.9813**
Body length, mm		Measured	*L*		1.854 (0.066)	− 0.003 to 0.085	
**Model 4**							
Total body water (Inverse), Ω	Serial-Individual	Calculated	TBWi*s2*	$(Xc \times R)/\sqrt[2]{{Xc^{2} + R^{2} }}$	− 3.091 (0.002)	− 3.720 to − 0.819	857.547
**Model 5**							
Total body water (Inverse), Ω	Serial-Individual	Calculated	TBWi*s2*	$(Xc \times R)/\sqrt[2]{{Xc^{2} + R^{2} }}$	− 4.215 (4.200E−5)	− 2.920 to − 1.057	**844.8685**
Body length, mm		Measured	*L*		1.861 (0.065)	− 0.003 to 0.085	
	**Proteins (%)**						
**Model 1**							
Resistance, Ω	Serial	Calculated	*R*	(*1/Y*)* × cos*(*ϕ*)	1.831 (0.069)	− 0.014 to 0.383	478.372
**Model 2**							
Reactance, Ω	Parallel	Calculated	*Xcp*	(1/*Y*)/*sin*(*− ϕ*)	1.942 (0.054)	− 0.001 to 0.163	479.966
**Model 3**							
Extra-cellular water (Inverse), Ω/mm	Serial-Individual	Calculated	ECWi*s*	*R*/*distance*	2.077 (0.039)	0.001 to 0.059	**481.980**
**Model 4**							
Intra-cellular water, Ω/mm	Parallel-Individual	Calculated	ICWi*p*	*Xcp/distance*	2.664 (0.009)	0.004 to 0.026	483.392
	**Minerals (%)**						
**Model 1**							
Intra-cellular water	Serial	Calculated	ICW*s2*	*Xc/R*	− 2.692 (0.008)	− 0.801 to − 0.123	− 62.263
**Model 2**							
Intra-cellular water	Serial	Calculated	ICW*s2*	*Xc/R*	− 2.701 (0.008)	− 0.616 to − 0.096	− **68.891**
Body weight		Measured	Wt		1.654 (0.100)	− 0.043 to 0.490	

The t-statistic, p-value and 95% confidence interval (95% CI) of the predictions show, respectively, the likelihood that the effect is different from zero, the probability that the effect can purely be assigned to chance, and the effect precision/size. The Akaike Information Criterion (AIC) was used for model selection and AIC cells in bold are the best fitting significant models (i.e., smaller values of the AIC were preferred). Distance in formulas is an interval between pairs of electrodes: widths in this schema.

#### Percent of total body protein

Significant positive effects on body protein percentage assessed by proximate composition analysis were found for in parallel obtained segmental BIA measures: reactance and total impedance, as well as a serially obtained segmental BIA measure: resistance, all adjusted for widths of segments (Supplementary Table S2). According to AIC, the prediction of body protein by these simple models did not improve if they also included body length or weight (Supplementary Table S3). Significant interaction effects of the adjusted in parallel and serially obtained reactance and resistance were also found for body protein, but according to AIC, the models were poorer fit compared to the simple models (Supplementary Table S3). The same was found when body length was added to the interaction models. According to AIC, the best fitting models were simple segmental BIA models of serially obtained resistance unadjusted (only approached significance in GLMM) and adjusted for widths of body segments (was significant in GLMM). Two other simple segmental BIA indicators, in parallel obtained reactance unadjusted (only approached significance in GLMM) and adjusted for widths of body segments (was significant in GLMM) were close in model fit (Supplementary Table S2). The best fitting models for protein-related effects from Supplementary Table S2 were presented in Table 2.

Serially obtained segmental BIA reactance and resistance, both adjusted for widths of segments and included in the same model, indicated significant negative and positive simple effects, respectively, on protein content. The same models with in parallel obtained segmental BIA reactance and resistance indicated significant effects in the opposite direction, specifically positive and negative simple effects, respectively, on protein content. Thus, quantity of proteins assessed locally or segmentally by serially obtained resistance (the best fitting) or in parallel obtained reactance (a less fitting) can represent the body protein with and without adjustment for widths of locally assessed segments and without accounting for the whole length or weight of the particular body as an individual trait.

#### Percent of total body ash/minerals

Significant negative effects on body ash percentage assessed by proximate composition analysis were found for serially obtained BIA measures: phase angle, reactance-to-resistance ratio, reactance adjusted for widths of segments, a product of reactance and resistance adjusted for widths of segments and with additional adjustment for total impedance, as well as for an in parallel obtained BIA measure: resistance adjusted for widths of segments (Supplementary Table S2). According to AIC, the prediction of body ash was improved if the simple BIA models also included body length or weight (best fitting models) as covariates (Supplementary Table S3).

Significant effects on body ash percentage were also found for various in serial and in parallel obtained BIA indicators when the indicators were added to models together (Supplementary Table S2). Serially obtained BIA reactance and resistance both included in the same model indicated significant opposite negative and positive simple effects, respectively, on ash content, while the same models with in parallel obtained BIA reactance and resistance indicated significant positive and negative simple effects, respectively, on ash content. However, the models had poorer fit compared to simple models, according to AIC. An improvement in fit closer to simple models was found when body length or body weight (most fitting models) was additionally included in these complex models. According to AIC, the best fitting models were a serially obtained reactance-to-resistance ratio with and without the effect adjusted for body weight. These models for ash-related effects from Supplementary Tables S2 and S3 were presented in Table 2. Thus, a relative quantity of ash assessed by the reactance-to-resistance ratio locally or segmentally can represent the total body ash after accounting for weight as a proxy of its distribution in the whole body.

#### Percent of total body fat

No significant effects on percentage of total body fat/lipids assessed by proximate composition analysis were found for segmental BIA measures.

#### The Fulton’s condition factor of fish ‘well-being’

No significant effects on the condition factor were obtained for segmental BIA measures.

### Whole body BIA relations to compartments in percent obtained by proximate composition analysis

#### Percent of total body moisture

Significant positive effects on body moisture percentage assessed by proximate composition analysis were found for ‘volumetric-based’ whole-body BIA models that used serially obtained measures of reactance (*distance*^*2*^/*Xc)* and total impedance (*distance*^*2*^*/Z*), and a parallelly obtained measure of resistance (*distance*^*2*^*/R*_*p*_) (Supplementary Table S5). According to AIC, the ‘volumetric-based’ whole-body BIA model with the parallelly obtained resistance (*distance*^*2*^*/R*_*p*_) was found to be the best fitting for percent body moisture obtained by proximate composition analysis (Table 3). Including body weight in the model improved the model fitting and its effect size but made the model non-significant.

**Table 3:** Best fitted effects of whole-body bioimpedance measures on body water/moisture, proteins, and ash/minerals (in percent).

Measures	Moisture (%)						
Name	Schema	Obtained	Symbol	Equation	t(p)	95% CI	AIC
**Model 1**							
Total body water, mm^2^/Ω	Parallel-empirical	Calculated	TBW*p*	*distance*^*2*^*/Rp*	2.217 (0.033)	0.020 to 0.441	**214.518**
**Model 2**							
Total body water, mm^2^/Ω	Parallel-empirical	Calculated	TBW*p*	*distance*^*2*^*/Rp*	1.993 (0.054)	− 0.003 to 0.380	210.326
Body Weight, kg		Measured	*Wt*		0.670 (0.507)	− 2.989 to 5.943	
	**Proteins (%)**						
**Model 1**							
Extra-cellular water (Inverse), Ω/mm	Parallel-individual	Calculated	ECWi*p*	*Rp/distance*	− 2.449 (0.019)	− 16.791 to − 1.593	**112.987**
**Model 2**							
Extra-cellular water (Inverse), Ω/mm	Parallel-individual	Calculated	ECWi*p*	*Rp/distance*	− 2.120 (0.041)	− 17.632 to − 0.400	111.572
Body Weight, kg		Measured	*Wt*		0.051 (0.960)	− 2.131 to 2.241	
	**Minerals (%)**						
**Model 1**							
Extra-cellular water (Inverse), Ω/mm	Parallel-individual	Calculated	ECWi*p*	*Rp/distance*	− 2.917 (0.006)	− 2.261 to − 0.408	− **12.681**
**Model 2**							
Extra-cellular water (Inverse), Ω/mm	Parallel-Individual	Calculated	ECWi*p*	*Rp/distance*	− 0.832 (0.411)	− 1.877 to 0.784	− 13.451
Body Weight, kg		Measured	*Wt*		1.493 (0.144)	− 0.090 to 0.591	

The t-statistic, p-value and 95% confidence interval (95% CI) of the predictions show, respectively, the likelihood that the effect is different from zero, the probability that the effect can purely be assigned to chance, and the effect precision/size. The Akaike Information Criterion (AIC) was used for model selection and AIC cells in bold are the best fitting significant models (i.e., smaller values of the AIC were preferred). Distance in formulas is an interval between pairs of electrodes: lengths in this schema.

#### Percent of total body protein

Significant negative effects on body protein percentage assessed by proximate composition analysis were found for an in parallel obtained whole-body BIA resistance adjusted for the distance between electrodes, serially obtained whole-body BIA phase angle, reactance-to-resistance ratio, and reactance with and without adjustment for the distance between the electrodes, as well as more complex whole-body BIA indicators: products of serially obtained reactance and resistance with and without adjustment for the distance between the electrodes, with additional adjustment for the serially obtained total impedance (Supplementary Table S5). According to AIC, the best fitting model was a simple whole-body model of parallelly obtained resistance adjusted for the distance between electrodes (*R*_*p*_/*distance*) (Table 3). Including body weight in the model only slightly improved the model fitting but decreased its effect size.

#### Percent of total body ash/minerals

Significant negative effects on total body ash percentage assessed by proximate composition analysis were found for an in parallel obtained whole-body BIA resistance with and without adjustment for the distance between electrodes, serially obtained whole-body BIA phase angle, reactance-to-resistance ratio, reactance with and without adjustment for the distance between electrodes, resistance with adjustment for distance between electrodes, as well as more complex whole-body BIA models: two with a product of serially obtained reactance and resistance with adjustment for the total impedance (with and without additional adjustment of each measure in the equations for the distance between electrodes) and one involving the serially obtained total impedance with adjustment for distance between electrodes (Supplementary Table S5). Significant positive effects on percent of total body ash were also found for ‘volumetric-based’ whole-body BIA models with a serially obtained reactance (*distance*^*2*^/*Xc*) and an in parallel obtained resistance (*distance*^*2*^*/R*_*p*_). According to AIC, the best fitting model was a simple whole-body BIA model of a parallelly obtained resistance adjusted for the distance between electrodes (*R*_*p*_/*distance*) (Table 3). Including body weight in the model only slightly improved the model fitting but decreased its effect size and made the model non-significant.

#### Percent of total body fat

No significant effects on total body fat/lipids percentages assessed by proximate composition analysis were found for whole-body BIA measures.

#### The Fulton’s condition factor of fish ‘well-being’

Whole-body BIA models with a serially obtained reactance and a product of serially obtained reactance and resistance, adjusted for the total impedance, significantly and positively predicted and was the best fitting model for the Fulton’s condition factor of fish ‘well-being’ (Supplementary Table S5).

### Other findings

Supplementary Tables S1 and S4 present predicting effects of all segmental and whole-body BIA measures on body weight, width, and length. According to AIC, serially obtained reactance to resistance ratio of segmental BIA was the best fitting model for predicting body weight and length, and a ‘volumetric-based’ model with the serially obtained reactance (*distance*^*2*^*/Xc*) of segmental BIA was the best fitting model for predicting body width that was used as distance in this BIA schema. In parallel obtained resistance of whole-body BIA adjusted to distance (i.e., to length in this schema) was the best fitting model for predicting body weight, and a ‘volumetric-based’ model with in parallel obtained reactance (*distance*^*2*^*/Xcp*) of whole-body BIA was the best fitting model for predicting body length. Body weight and widths of segments were not significantly related to percent of body moisture (water), protein, ash, and fat. Body length was only significantly related to percent of body ash (Supplementary Tables S2 and S5).

## Discussion

For the present study, fish was selected as a biological model to validate BIA equations in predicting proximate body components associated with hydration status such as water, proteins including dissolved (colloid) fraction, and minerals including non-osseous fraction and associated with nutrition status such as fat, all obtained directly by physicochemical methods. Only these direct measures adjusted for inter-individual differences in weight (i.e., represented as percent of body water, proteins, minerals, and fat) were used as references to validate best fitting BIA equations for their future use in indirect assessment of body components in humans. These BIA equations were approved in the study with respect to the principal predictive values of impedance and its resistance and reactance components, as well as their various ratios and products assessed in series or in parallel, using whole-body and segmental BIA schemas that should be common across different biological species, but without inclusion of specific regression constants that should differ between different biological species^20^. The length of body, distance between electrodes, and weight as additional parameters of individual differences, or individual traits, were assessed for their impact in improvement of the predictive value of the BIA measures.

Findings of the present study confirmed in non-human biological subjects (*Cyprinus rex cyprinorum*) previous considerations that models containing BIA measures obtained from whole-body derivations could predict total body content of water/moisture, protein, and ash/mineral percentages, but not fat, assessed by a similar proximate physicochemical composition analysis. This confirmed the reliability of the design of the present study including the BIA measurement procedure and the lethal (physicochemical) method for the proximate composition analysis. Moreover, the study showed that models containing BIA measures obtained from segmental impedance readings could also predict these proximate measures of body composition. However, best fitting models predicting the body compartments were different for BIA measures from segmental versus whole-body BIA readings. The main difference was related to different electrical compensation schemes used to obtained BIA measures for inclusion in prediction models: serial for segmental and parallel for the whole-body BIA readings.

Another difference was that, in contrast to segmental models, in the whole-body BIA schema, the total body water content was better predicted by the BIA measures obtained using a ‘volumetric-based’ model rather than models unadjusted for body volume as a conductor. The ‘volumetric-based’ model, the most exploited model of body composition assessment, uses adjustment of individual body length as a proxy of distance that current runs between electrodes to determine BIA measures (*distance*^*2*^/*R*) and then additionally to calculate population-derived regression coefficients. In contrast, in the segmental BIA schema, the equations combining measures of resistance and reactance (e.g., as their ratio or their product) with or without adjustment for inter-electrode distances (i.e., width in the present case), as in classical BIVA models (*R/distance*), or with adjustment for the total bioimpedance variable, were the best predictors of the total body water content in this study. Length of body as an additional parameter of individual differences, or the individual trait, was found to improve the predictive value of the total water percentage by the segmental or local BIA if it was added in the regression formula.

With respect to between-subject variation in the body hydration status, the association of body moisture decrease with an increase in both serially obtained resistance and reactance bioimpedance measures indicated that the dehydration was probably related to (or was interpreted as) a mechanism of water transfer from ECW (water decrease) to ICW (water increase). Since reactance (*Xc*) is related to the dielectric properties, it is assumed that ICW should linearly and positively be correlated with the reactance (*Xc*), while resistance (*R*) should linearly and negatively be correlated with ECW^16^. In addition, this purported ECW to ICW distribution shift was confirmed by a significant relationship of lower body moisture with a higher value for the product of the resistance and reactance adjusted for total bioimpedance. However, best fitting models related to higher reactance to resistance ratio and higher absolute reactance could indicate a predominant effect of absolute ICW increase (i.e., BIA reactance increase) compared with ECW decrease (i.e., BIA resistance increase). This finding corresponds with a study that showed the effect of greater osmolarity or dehydration in response to exercise with the increase in *Xc*/*L* (i.e., an increase in ICW or intracellular fluid)^14^. Indeed, ICW has a higher resistivity than ECW primarily due to the high concentration of dissolved protein (i.e., high viscosity), and thus ion movement in response to current should be more inhibited during dehydration^16^. Both in parallel and in series obtained resistance and reactance in both whole-body and segmental schemas were similarly related to body moisture proposing that they detected a hydration status of a similar origin.

In the whole-body BIA schema, percent of body proteins and ash/minerals decreases were better predicted by an increase in parallel obtained resistance (i.e., a purported ECW decrease) adjusted for only inter-electrode distance, as in BIVA models (i.e., length in the present case). In the segmental BIA obtained in series schema, the protein content decrease was better predicted by a decrease of a resistance measure (i.e., a putative ECW increase) without adjustment to individual difference in body weight or length, but the ash/minerals content decrease was better predicted by an increase of a reactance measure (i.e., a putative ICW increase) with adjustment to individual difference in body weight. Moreover, segmental schema models with both serially obtained resistance and reactance in the formulas showed that both protein and ash/minerals decreases were associated with decreasing resistance and increasing reactance, interpreted as a parallel increase in ECW and ICW.

In contrast, the same segmental schema models with parallelly obtained resistance and reactance in the formulas showed that protein and ash/minerals decreases were associated with increasing resistance and decreasing reactance, interpreted as a parallel decrease in ECW and ICW. This proposes a distinct origin of these compartments and their correspondence to hydration status detected by serially and parallelly obtained electrical compensation schemas. For example, ICW has a higher specific resistance than ECW primarily due to the high concentration of dissolved protein, which dramatically impedes ion movement associated with water resistivities regulated by non-osseous minerals presented in fluids as electrolytes: mainly chloride for ECW and mainly potassium for ICW^16^. Serially detected protein and minerals predominantly in the segmental models could indicate the decrease in body fluids with an increase in concentrations of electrolytes and dissolved proteins as in hyper-osmolal dehydration that was expected in the current design. Parallelly detected protein and minerals predominantly in the whole-body models could indicate the decrease in body fluids with a decrease in concentrations of electrolytes and dissolved proteins mimicking eu-osmolal dehydration that was not expected in the current design. Thus, the parallel schema might assess percent of electrolytes and dissolved proteins that were concentrated intracellularly. This corresponds to the proposal that bioimpedance measured at 50 kHz current obtained by a serial schema primarily reflects the ECW space, but a parallel bioimpedance model is more sensitive to changes in ICW^12^. Moreover, in contrast to body moisture, ash/minerals and protein contents were not only related to separate BIA resistance or reactance changes but were also related to their simultaneous independent changes (ash/minerals content) or to their simultaneous independent and combined changes (protein content). Thus, the use of two (in series and in parallel) electrical compensation schemas could allow for measuring the osmolality (hyper-, hypo- or iso-osmolality) of different origin (associated with dissolved protein or electrolytes), but not the hydration status (hyper-, hypo- or iso-hydration) separately in the ECW and ICW compartments by bioimpedance measures using the single 50 kHz frequency of electrical current. The findings of different relationships of body moisture, protein, and ash/minerals to the same resistance and reactance measures of total impedance suggest that this single frequency electrical schema can separately predict the hydration and osmolality status associated with TBW, as well as its ECW and ICW compartments using the segmental BIA model. This also confirms previous findings suggesting that electrolyte balance influences BIA measurements independently of fluid changes^12,31^.

One more difference between the schemas was related to a significant prediction of body ‘well-being’ by models of the whole-body but not the segmental BIA schema. A whole-body BIA model with serially obtained reactance positively predicted and best fit the Fulton’s condition factor of fish ‘well-being’. It can be interpreted as more ICW should correspond to higher ‘well-being’ or better nutritional condition^50^. Both segmental and whole-body BIA readings in response to the single 50 kHz frequency did not predict the fat or lipid compartment of the body composition. Poor correlations between the similar procedure of BIA measurement and fat/lipid content have been frequently observed in fish and are proposed to be related to a higher electrical resistivity of fat mass to the current at this frequency compared with other types of tissues^23,51^.

Additionally, most whole-body BIA models included the total length of the body in their equations, and their ability to accurately predict the body components may simply be related to the adjustment of BIA measures for body length or length squared^17^. While this proposal may be true for absolute measures of water, protein, and ash/minerals (data not presented), and to some extent (according to small improvements in information criteria) for percent of total water and minerals in whole-body models, and for segmental BIA models with statistical adjustment for body length or weight, the prediction of percent of total protein by segmental BIA models was not dependent on these variables. Moreover, compared with models developed using only width (i.e., inter-electrode distance), body length, or body weight data alone, the segmental and whole-body BIA models were consistently significant and explained more variability in laboratory-derived estimates of percent moisture, protein, and ash/minerals content as assessed by AIC.

### Limitations

The present study did not include an assessment of the relative contribution of each segment in ventral and dorsal surfaces (i.e., electrode location and its type) on the predictive ability of the BIA models. The validity of BIA models also invariably relies on the amount of contrast in the proximate composition of the studied samples that was not specially manipulated in the present study. To adequately assess the ability of the BIA models to predict body compartments associated with hydration and osmolality status, future study should include a wider range of physiological states within a particular population and information on each of these states with cross-sectional (e.g., from hypo- to over-hydration combined or non-combined with osmolality changes) or repeated measures (e.g., de- and re-hydration with or without osmolality changes) design.

## Conclusion

Since BIA works very similarly for a wide range of vertebrates from humans to fish^20^, the latter was used in this study as a model for the comparison of different BIA measures and equations to predict between-subject variance in proximate body measures of hydration status (i.e., percent of total body moisture, proteins, and minerals) obtained by physicochemical analyses after destruction of the fish body. This approach bypasses shortcomings of most non-lethal or in-vivo reference methods applied in human subjects affecting the precision of the related body compartment models.

In summary, while the majority of studies of the whole-body BIA use ‘volumetric-based’ calculations of electrical variables with proposal of either serial or parallel electrical circuits in biological tissues, the present study found that in parallel obtained whole-body BIA measures predicted better proximate composition measures. Moreover, utilizing other BIA equations obtained by a segmental or local BIA schema using in series electrical compensation schema, specifically the ratio of the two vector components (*Xc/R*) and their product adjusted to the total bioimpedance ([Xc*R]/Z) both corrected for body length, the ratio of the two vector components (*Xc/R*) corrected for body weight, and the resistance adjusted to distance between electrodes (*R/distance*) with independence of inter-individual variability in weight and length, were found to be more valid in the prediction of components associated with hydration status, specifically body water, minerals, and protein content, respectively. The latter equations are often missed in analyses compared with the ‘volumetric-based’ formula, and this may bring contention regarding the utility of the BIA technique. Moreover, their prediction by different equations corrected or uncorrected for body weight and length was probably related to different distribution of the hydration components between ICW and ECW spaces affecting not only their hydration, but separately also their osmolality status. Some of the current findings showed that the application of both in series and in parallel electrical compensation schemas for BIA measurement at 50 kHz frequency of electrical current could guarantee that ECW differences do not corrupt the ICW and vice versa in assumed osmolality status assessment (electrolyte balance coupled with dissolved protein level affecting, respectively, osmotic and oncotic pressures), but probably were not important for hydration status (water balance) assessment as two separate and relatively independent targets of the homeostatic regulation. Thus, the results of the present comparative study investigating various BIA modelling and measurement techniques suggest that various segmental/local and whole-body BIA models are capable of predicting the proximate content of the biological subject with different precision. However, findings of indirect bioimpedance-derived measures of hydration and osmolality homeostasis obtained in fish should be transferred to humans after optimizing applicability of respective equations to bioimpedance measures obtained at different segmental and local anatomical portions of the human body^52^. Moreover, validity of these BIA models should be confirmed while controlling for potential confounding factors before implementation of the best techniques for the mathematical treatment of BIA data in practice. Formulas for predicting absolute values for these body compartments in the assessment of nutrition status will require adjustment for regression constants that should differ between animal (e.g., fish) and human populations, an objective for a future study.

## Supplementary Information

Supplementary Information.

## Data Availability

The datasets generated during and/or analysed during the current study are available from the corresponding author on reasonable request.
