# Supplementary material for: Making the choice between bioelectrical impedance measures for body hydration status assessment
Source: Sci Rep. 2021 Apr 8;11:7685. doi: 10.1038/s41598-021-87253-4 (PMC8032770; doi:10.1038/s41598-021-87253-4)
Supplement: Supplementary file 1 — Supplementary Information. [file 41598_2021_87253_MOESM1_ESM.pdf]

Supplementary materials

Title: Making the choice between bioelectrical impedance measures for body hydration status assessment

Authors:

Dmitry M. Davydov [a, *], PhD, MD

Andrey Boev [b]

Stas Gorbunov [b]

Affiliations:

[a] Laboratory of Neuroimmunopathology, Institute of General Pathology and Pathophysiology, Russian Academy of Sciences, 8 Baltiyskaia ul., Moscow, 125315, Russia. Email: d.m.davydov@gmail.com

[b] Departments of Research and Development, Aura Devices Inc., 1013 Centre Road, Suite 403-B, Wilmington, Delaware, 19805, USA; Emails: ab@auraband.io; sg@auraband.io

*Correspondence to Dmitry M. Davydov, Laboratory of Neuroimmunopathology, Institute of General Pathology and Pathophysiology, Russian Academy of Sciences, 8 Baltiyskaia ul., Moscow, 125315, Russia; Phone: 7 (495) 496 5234; Fax: 7 (495) 601 2366; E-mail: d.m.davydov@gmail.com

Table 1S. Predicting main effects of segmental bioimpedance (BIA) measures on body weight, width, and length in percent to body weight. The t-statistic, p-value and 95% confidence interval (95% CI) of the predictions show, respectively, the likelihood that the effect is different from zero, the probability that the effect can purely be assigned to chance, and the effect precision/size. The Akaike Information Criterion (AIC) was used for model selection and AIC cells in bold are the best fitting significant models (i.e., smaller values of the AIC were preferred). Distance in formulas is an interval between pairs of electrodes: widths in segmental schema and body lengths in whole-body schema. [1] - measured by RLC analyser E7-25.

Measures					Weight			Width			Length		
Name	Schema	Obtained	Symbol	Equation	t(p)	95% CI	AIC	t(p)	95% CI	AIC	t(p)	95% CI	AIC
Conductance module	Parallel	Measured [1]	*Y*		NS			-25.643 (0.0E0)	-4.346 to -3.724	1375.778	NS		
Phase angle	Serial	Measured [1]	ϕ		-3.723 (2.74E-4)	-0.018 to -0.006	-20.772	-5.142 (7.95E-7)	-0.709 to -0.316	1369.777	-3.665 (3.37E-4)	-1.951 to -0.584	1529.468
Resistance	Serial	Calculated	*R*	$(1/Y) * cos(\phi)$	NS			-8.236 (6.33E-14)	-10.089 to -6.186	1370.722	NS		
Reactance	Serial	Calculated	*Xc*	$(1/Y) * sin(\phi)$	-2.293 (0.023)	-0.220 to -0.016	-15.543	-3.843 (1.76E-4)	-27.849 to -8.940	1361.901	NS		
Total body water	Serial-Empirical	Calculated	TBW*s*	$distance^2/R$	4.177 (4.90E-5)	0.020 to 0.057	-17.142	25.317 (0.0E0)	14.621 to 17.096	1144.44	3.169 (0.002)	1.615 to 6.961	1529.73
Intra-cellular water (Inverse)	Serial-Empirical	Calculated	ICW*s*	$distance^2/Xc$	4.413 (1.90E-5)	0.013 to 0.033	-18.635	**34.350 (0.0E0)**	**7.708 to 8.649**	**1116.443**	3.588 (4.45E-4)	1.125 to 3.881	1529.266
Extra-cellular water	Serial-Empirical	Calculated	ECW*s*	TBW*s*- ICW*s*	-4.374 (2.20E-5)	-0.069 to -0.026	-21.961	-20.569 (0.0E0)	-13.294 to -10.964	1163.157	NS		
Intra-cellular water	Serial	Calculated	ICW*s2*	Xc/R	**-3.558 (4.93E-4)**	**-0.844 to -0.242**	**-27.896**	-5.082 (1.00E-6)	-33.082 to -14.565	1362.953	**-3.529 (0.001)**	**-88.356 to -24.944**	**1522.235**
Intra-cellular water	Serial-Individual	Calculated	ICWi*s*	Xc/distance	-4.506 (1.30E-5)	-0.030 to -0.012	-12.744	-2.626 (0.009)	-6.159 to -0.871	1354.73	-2.247 (0.026)	-2.763 to -0.178	1534.259
Extra-cellular water (Inverse)	Serial-Individual	Calculated	ECWi*s*	R/distance	-1.770 (0.079)	-0.015 to 0.001	-9.526	-5.124 (8.630E-7)	-7.158 to -3.175	1355.567	NS		
Total body water (Inverse)	Serial-Individual	Calculated	TBWi*s*	$\sqrt[2]{Xc^2 + R^2}$	NS			-7.492 (4.52E-12)	-9.389 to -5.471	1369.672	NS		
Resistance	Parallel	Calculated	*Rp*	$(1/Y) / cos(-\phi)$	NS			-6.028 (1.23E-8)	-8.627 to -4.369	1369.041	NS		
Reactance	Parallel	Calculated	*Xcp*	$(1/Y) / sin(-\phi)$	2.459 (0.015)	0.004 to 0.037	-11.961	NS			2.167 (0.032)	0.227 to 3.911	1532.939
Total body water	Parallel-Empirical	Calculated	TBW*p*	$distance^2/Rp$	4.331 (2.60E-5)	0.028 to 0.074	-18.344	34.386 (0.0E0)	19.421 to 21.788	1124.429	3.361 (0.001)	2.333 to 8.983	1528.797
Intra-cellular water (Inverse)	Parallel-Empirical	Calculated	ICW*p*	$distance^2/Xcp$	3.853 (1.69E-4)	0.042 to 0.129	-17.572	19.241 (0.0E0)	33.293 to 40.909	1178.148	2.854 (0.005)	2.931 to 16.100	1528.842
Extra-cellular water	Parallel-Empirical	Calculated	ECW*p*	TBW*p*- ICW*p*	4.442 (1.70E-5)	0.062 to 0.161	-21.399	27.081 (0.0E0)	37.924 to 43.891	1149.299	NS		
Intra-cellular water	Parallel	Calculated	ICW*p2*	Xcp/Rp	3.840 (1.77E-4)	0.040 to 0.126	-24.438	4.472 (1.50E-5)	1.612 to 4.161	1364.353	3.644 (3.63E-4)	4.096 to 13.789	1525.446
Intra-cellular water	Parallel-Individual	Calculated	ICWi*p*	$Xcp/distance$	NS			-5.301 (3.819E-7)	-2.617 to -1.196	1362.971	NS		
Extra-cellular water (Inverse)	Parallel-Individual	Calculated	ECWi*p*	$Rp/distance$	-2.791 (0.006)	-0.012 to -0.002	-9.814	-3.592 (4.38E-4)	-5.665 to -1.645	1359.407	NS		
Total body water (Inverse)	Parallel-Individual	Calculated	TBWi*p*	$\sqrt[2]{Xcp^2 + Rp^2}$	1.792 (0.075)	-0.002 to 0.032	-11.028	NS			1.806 (0.073)	-0.188 to 4.206	1533.619
Total body water (Inverse)	Serial-Individual	Calculated	TBWi*s2*	$(Xc*R)/\sqrt[2]{Xc^2 + R^2}$	NS			-4.509 (1.30E-5)	-32.720 to -12.786	1360.888	NS		
Total body water	Serial-Empirical	Calculated	TBW*s2*	$distance^2/\sqrt[2]{Xc^2 + R^2}$	4.263 (3.50E-5)	0.024 to 0.065	-17.751	29.320 (0.0E0)	16.903 to 19.345	1133.06	3.270 (0.001)	1.954 to 7.917	1529.257
Total body water (Inverse)	Serial-Individual	Calculated	TBWi*s3*	$\sqrt[2]{ICWis^2 + ECWis^2}$	-2.200 (0.029)	-0.014 to -0.001	-9.664	-4.352 (2.40E-5)	-6.391 to -2.401	1357.1	NS		
Total body water (Inverse)	Parallel-Individual	Calculated	TBWi*p2*	$\sqrt[2]{ICWip^2 + ECWip^2}$	NS			-5.214 (5.71E-7)	-2.337 to -1.053	1362.273	NS		
Total body water (Inverse)	Serial-Individual	Calculated	TBWi*s4*	$\frac{10 * (ICWis * ECWis)}{\sqrt[2]{ICWis^2 + ECWis^2}}$	-3.661 (3.42E-4)	-0.004 to -0.001	-8.095	-3.284 (0.001)	-1.690 to -0.421	1360.144	-2.022 (0.045)	-0.333 to -0.004	1538.566
Body Weight		Measured						5.368 (4.20E-5)	17.949 to 41.032	1348.041	5.961 (1.20E-5)	70.594 to 147.446	1353.887
Body Width (average)		Measured			7.116 (1.000E-6)	0.014 to 0.026	-12.796					4.683 (1.850E-4)	1.318 to 3.464
Body Length		Measured			7.778 (3.650E-7)	0.005 to 0.008	-7.876	4.866 (1.240E-4)	0.114 to 0.287	1365.373			

Table 2S. Predicting main effects of segmental BIA measures on body water/moisture, proteins, and ash/minerals in percent to body weight. See explanation of abbreviations and marks in legend to Table 1S.

Measures					Moisture			Proteins			Ash		
Name	Schema	Obtained	Symbol	Equation	t(p)	95% CI	AIC	t(p)	95% CI	AIC	t(p)	95% CI	AIC
Conductance module	Parallel	Measured [1]	*Y*		NS			NS			NS		
Phase angle	Serial	Measured [1]	ϕ		-1.888 (0.061)	-0.207 to 0.005	862.961	NS			-2.583 (0.011)	-0.017 to -0.002	-53.604
Resistance	Serial	Calculated	*R*	$(1/Y) * cos(\phi)$	-1.729 (0.086)	-1.444 to 0.096	860.485	1.831 (0.069)	-0.014 to 0.383	478.372	NS		
Reactance	Serial	Calculated	*Xc*	$(1/Y) * sin(\phi)$	-3.312 (0.001)	-3.282 to-0.830	857.459	NS			NS		
Total body water	Serial-Empirical	Calculated	TBW*s*	$distance^2/R$	NS			NS			NS		
Intra-cellular water (Inverse)	Serial-Empirical	Calculated	ICW*s*	$distance^2/Xc$	NS			NS			1.764 (0.080)	-0.001 to 0.017	-44.804
Extra-cellular water	Serial-Empirical	Calculated	ECW*s*	TBW*s*- ICW*s*	NS			NS			NS		
Intra-cellular water	Serial	Calculated	ICW*s2*	Xc/R	**-2.088 (0.038)**	**-9.918 to -0.276**	**854.478**	NS			**-2.692 (0.008)**	**-0.801 to -0.123**	**-62.263**
Intra-cellular water	Serial-Individual	Calculated	ICWi*s*	$Xc/distance$	-4.522 (1.2E-5)	-0.378 to -0.148	861.999	NS			-3.354 (0.001)	-0.023 to -0.006	-46.079
Extra-cellular water (Inverse)	Serial-Individual	Calculated	ECWi*s*	$R/distance$	-2.780 (0.006)	-0.219 to -0.037	863.731	**2.077 (0.039)**	**0.001 to 0.059**	**481.980**	NS		
Total body water (Inverse)	Serial-Individual	Calculated	TBWi*s*	$\sqrt[2]{Xc^2 + R^2}$	-2.133 (0.034)	-1.357 to -0.052	860.332	1.706 (0.090)	-0.024 to 0.323	478.747	NS		
Resistance	Parallel	Calculated	*Rp*	$(1/Y) / cos(-\phi)$	-2.596 (0.010)	-1.234 to -0.168	860.163	NS			NS		
Reactance	Parallel	Calculated	*Xcp*	$(1/Y) / sin(-\phi)$	NS			1.942 (0.054)	-0.001 to 0.163	479.966	NS		
Total body water	Parallel-Empirical	Calculated	TBW*p*	$distance^2/Rp$	NS			NS			NS		
Intra-cellular water (Inverse)	Parallel-Empirical	Calculated	ICW*p*	$distance^2/Xcp$	NS			NS			NS		
Extra-cellular water	Parallel-Empirical	Calculated	ECW*p*	TBW*p*- ICW*p*	NS			NS			NS		
Intra-cellular water	Parallel	Calculated	ICW*p2*	Xcp/Rp	NS			NS			1.700 (0.091)	-0.008 to 0.110	-50.133
Intra-cellular water	Parallel-Individual	Calculated	ICWi*p*	$Xcp/distance$	-2.065 (0.041)	-0.089 to -0.002	865.841	2.664 (0.009)	0.004 to 0.026	483.392	NS		
Extra-cellular water (Inverse)	Parallel-Individual	Calculated	ECWi*p*	$Rp/distance$	-3.945 (1.20E-4)	-0.161 to -0.054	863.922	1.738 (0.084)	-0.002 to 0.039	482.854	-2.154 (0.033)	-0.010 to -4.22E-4	-43.733
Total body water (Inverse)	Parallel-Individual	Calculated	TBWi*p*	$\sqrt[2]{Xcp^2 + Rp^2}$	NS			1.940 (0.054)	-0.001 to 0.143	480.251	NS		
Total body water (Inverse)	Serial-Individual	Calculated	TBWi*s2*	$(Xc*R)/\sqrt[2]{Xc^2 + R^2}$	-3.091 (0.002)	-3.720 to -0.819	857.547	NS			NS		
Total body water	Serial-Empirical	Calculated	TBW*s2*	$distance^2/\sqrt[2]{Xc^2 + R^2}$	NS			NS			NS		
Total body water (Inverse)	Serial-Individual	Calculated	TBWi*s3*	$\sqrt[2]{ICWis^2 + ECWis^2}$	-3.330 (0.001)	-0.190 to -0.049	863.807	1.900 (0.059)	-0.001 to 0.049	482.400	-1.681 (0.095)	-0.011 to 0.001	-43.709
Total body water (Inverse)	Parallel-Individual	Calculated	TBWi*p2*	$\sqrt[2]{ICWip^2 + ECWip^2}$	-2.452 (0.015)	-0.079 to -0.009	865.884	2.471 (0.015)	0.002 to 0.022	483.777	NS		
Total body water (Inverse)	Serial-Individual	Calculated	TBWi*s4*	$\frac{10 * (ICWis * ECWis)}{\sqrt[2]{ICWis^2 + ECWis^2}}$	-4.195 (4.5E-5)	-0.045 to -0.016	866.402	NS			-2.568 (0.011)	-0.003 to-3.63E-4	-41.467
Body Weight		Measured			NS			NS			1.838 (0.083)	-0.039 to 0.587	-63.481
Body Width (average)		Measured			NS			NS			1.820 (0.085)	-0.001 to 0.016	-40.319
Body Length		Measured			1.852 (0.080)	-0.006 to 0.089	849.371	NS			2.311 (0.033)	2.110E-4 to 0.004	-58.134

Table 3S. Predicting two- and three-factor simple and interaction effects of segmental BIA measures on body water/moisture, proteins, and ash/minerals in percent to body weight. See explanation of abbreviations and marks in legend to Table 1S.

Measures		Moisture					Proteins					Ash		
Models	Symbols	t(p)	95% CI	AIC	Models	Symbols	t(p)	95% CI	AIC	Models	Symbols	t(p)	95% CI	AIC
Model 1				864.956	*Model 1*				487.4253	*Model 1*				-60.371
Conductance module	*Y*	1.666 (0.098)	-0.017 to 0.197		Intra-cellular water	ICWi*s*	-2.315 (0.022)	-1.094 to -0.087		Phase angle	ϕ	-2.593 (0.010)	-0.013 to -0.002	
Phase angle	ϕ	-2.091 (0.038)	-0.227 to -0.006		Extra-cellular water	ECWi*s*	2.558 (0.011)	0.048 to 0.371		Body Weight	*Wt*	1.668 (0.097)	-0.042 to 0.493	
Model 2				847.0097	Interaction	ICWi*s**ECWi*s*	2.316 (0.022)	0.003 to 0.041		*Model 2*				-56.907
Resistance	*R*	-2.670 (0.008)	-1.339 to -0.200		*Model 2*				490.4123	Intra-cellular water	ICWi*s*	-3.227 (0.002)	-0.014 to -0.003	
Body Length	*L*	1.889 (0.061)	-0.002 to 0.086		Intra-cellular water	ICWi*s*	-1.724 (0.087)	-0.892 to 0.061		Body Weight	*Wt*	1.814 (0.072)	-0.024 to 0.554	
Model 3				846.9917	Extra-cellular water	ECWi*s*	1.917 (0.057)	-0.005 to 0.314		***Model 3***				**-68.891**
Resistance	R_p	-3.600 (4.270E-4)	-1.064 to -0.310		Interaction	ICWi*s**ECWi*s*	1.979 (0.050)	2.99E-5 to 0.032		Intra-cellular water	ICW*s2*	-2.701 (0.008)	-0.616 to -0.096	
Body Length	*L*	1.880 (0.062)	-0.002 to 0.086		Body Length	*L*	0.993 (0.332)	-0.007 to 0.022		Body Weight	*Wt*	1.654 (0.100)	-0.043 to 0.490	
Model 4				**844.9813**	*Model 3*				495.4253	*Model 4*				-52.504
Reactance	*Xc*	-4.159 (5.200E-5)	-2.583 to -0.919		Intra-cellular water	ICWi*p*	2.158 (0.032)	0.004 to 0.087		Total body water	$TBWi_{s4}$	-2.425 (0.016)	-0.002 to -1.740E-4	
Body Length	*L*	1.854 (0.066)	-0.003 to 0.085		Extra-cellular water	ECWi*p*	-2.334 (0.021)	-0.253 to -0.021		Body Weight	*Wt*	1.822 (0.070)	-0.022 to 0.557	
Model 5				849.0913	Interaction	ICWi*p**ECWi*p*	2.617 (0.010)	0.001 to 0.005		*Model 5*				-45.9085
Reactance	Xc_p	-2.110 (0.036)	-0.522 to -0.017		*Model 4*				498.0291	Conductance module	*/Y/*	2.110 (0.036)	3.340E-4 to 0.10	
Body Length	*L*	1.927 (0.056)	-0.001 to 0.087		Intra-cellular water	ICWi*p*	1.423 (0.157)	-0.012 to 0.072		Phase angle	ϕ	-2.855 (0.005)	-0.018 to -0.003	
Model 6				849.6587	Extra-cellular water	ECWi*p*	-1.829 (0.069)	-0.192 to 0.007		*Model 6*				-52.175
Intra-cellular water	ICWi*s*	-5.168 (7.104E-7)	-0.283 to -0.126		Interaction	ICWi*p**ECWi*p*	2.904 (0.004)	0.001 to 0.004		Conductance module	*/Y/*	1.794 (0.075)	-3.850E-4 to 0.008	
Body Length	*L*	1.837 (0.068)	-0.003 to 0.085		Body Length	*L*	1.017 (0.311)	-0.007 to 0.023		Phase angle	ϕ	-2.804 (0.006)	-0.014 to -0.002	
Model 7				852.6848	*Model 5*				485.2908	Body Weight	*Wt*	1.620 (0.107)	-0.049 to 0.491	
Intra-cellular water	$ICWi_p$	-2.507 (0.013)	-0.082 to -0.010		Total body water	TBWi*p2*	2.450 (0.015)	0.002 to 0.023		*Model 7*				-54.263
Body Length	*L*	1.872 (0.063)	-0.002 to 0.086		Body Length	*L*	1.090 (0.277)	-0.007 to 0.023		Resistance	*R*	3.511 (0.001)	0.113 to 0.404	
Model 8				850.951						Reactance	*Xc*	-4.024 (8.900E-5)	-0.744 to -0.254	
Extra-cellular water	ECWi*s*	-2.897 (0.004)	-0.185 to -0.035							*Model 8*				-61.185
Body Length	*L*	1.853 (0.066)	-0.003 to 0.086							Resistance	*R*	2.720 (0.007)	0.049 to 0.310	
Model 9				851.3468						Reactance	*Xc*	-2.857 (0.005)	-0.579 to -0.106	
Extra-cellular water	$ECWi_p$	-4.142 (5.60E-5)	-0.131 to -0.046							Body Weight	*Wt*	1.618 (0.108)	-0.051 to 0.512	
Body Length	*L*	1.846 (0.067)	-0.003 to 0.085							*Model 9*				-47.1814
Model 10				846.9854						Resistance	R_p	-2.886 (0.004)	-0.259 to -0.048	
Total body water	TBWi*s*	-3.122 (0.002)	-1.205 to -0.271							Reactance	Xc_p	2.436 (0.016)	0.014 to 0.133	
Body Length	*L*	1.885 (0.061)	-0.002 to 0.086							*Model 10*				-54.702
Model 11				851.1288						Resistance	R_p	-2.257 (0.025)	-0.163 to -0.011	
Total body water	TBWi*s3*	-3.480 (0.001)	-0.157 to -0.043							Reactance	Xc_p	1.883 (0.061)	-0.002 to 0.085	
Body Length	*L*	1.849 (0.066)	-0.003 to 0.086							Body Weight	*Wt*	1.720 (0.087)	-0.036 to 0.518	
Model 12				**844.8685**						*Model 11*				-46.3861
Total body water	TBWi*s2*	-4.215 (4.200E-5)	-2.920 to -1.057							Intra-cellular water	ICW*s*	2.438 (0.016)	0.013 to 0.123	
Body Length	*L*	1.861 (0.065)	-0.003 to 0.085							Total body water	TBW*s*	-2.115 (0.036)	-0.240 to -0.008	
Model 13				852.8275						*Model 12*				-55.21
Total body water	TBWi*p2*	-2.854 (0.005)	-0.072 to -0.013							Intra-cellular water	ICW*s*	2.070 (0.040)	0.002 to 0.101	

Body Length	*L*	1.868 (0.064)	-0.002 to 0.086	
Model 14				853.9819
Total body water	TBWi*s4*	-4.640 (7.00E-6)	-0.034 to -0.014	
Body Length	*L*	1.840 (0.068)	-0.003 to 0.085	

Total body water	TBW*s*	-1.866 (0.064)	-0.210 to 0.006	
Body Weight	*Wt*	1.714 (0.088)	-0.038 to 0.539	
Model 13				-51.0932
Intra-cellular water	ICW*p*	-2.372 (0.019)	-0.719 to -0.066	
Total body water	TBW*p*	2.683 (0.008)	0.056 to 0.370	
Model 14				-60.351
Intra-cellular water	ICW*p*	-2.100 (0.037)	-0.633 to -0.019	
Total body water	TBW*p*	2.305 (0.023)	0.024 to 0.309	
Body Weight	*Wt*	1.738 (0.084)	-0.035 to 0.540	
Model 15				-42.4787
Intra-cellular water	ICWi*s*	-2.732 (0.007)	-0.102 to -0.016	
Extra-cellular water	ECWi*s*	1.928 (0.056)	-0.001 to 0.059	
Model 16				-51.867
Intra-cellular water	ICWi*s*	-2.117 (0.036)	-0.076 to -0.003	
Extra-cellular water	ECWi*s*	1.555 (0.122)	-0.005 to 0.045	
Body Weight	*Wt*	1.750 (0.082)	-0.033 to 0.540	

Table 4S. Predicting main effects of whole-body BIA measures on body weight and length. See explanation of abbreviations and marks in legend to Table 1S.

Measures				Weight			Length		
Name	Schema	Obtained	Symbol	t(p)	95% CI	AIC	t(p)	95% CI	AIC
Conductance module	Parallel	Measured	*Y*	1.908 (0.064)	-0.003 to 0.095	2.603	1.723 (0.093)	-0.693 to 8.623	354.169
Phase angle	Parallel	Measured	ϕ	NS			NS		
Resistance	Serial	Calculated	*R*	-1.763 (0.086)	-0.313 to 0.022	0.193	NS		
Reactance	Serial	Calculated	*Xc*	-3.952 (3.26E-4)	-0.475 to -0.153	-2.279	-3.475 (0.001)	-60.651 to -15.999	347.627
Total body water	Serial-Empirical	Calculated	TBW*s*	3.720 (0.001)	0.013 to 0.043	-4.421	4.142 (1.85E-4)	1.650 to 4.805	343.893
Intra-cellular water (Inverse)	Serial-Empirical	Calculated	ICW*s*	3.097 (0.004)	0.002 to 0.011	3.024	3.315 (0.002)	0.288 to 1.190	353.593
Extra-cellular water	Serial-Empirical	Calculated	ECW*s*	-2.822 (0.008)	-0.014 to -0.002	4.425	-3.065 (0.004)	-1.406 to -0.257	355.56
Intra-cellular water	Serial	Calculated	ICW*s2*	NS			-1.730 (0.092)	-90.055 to 7.073	349.727
Intra-cellular water	Serial-Individual	Calculated	ICWi*s*	-6.844 (4.004E-8)	-0.832 to -0.452	-5.497	-9.230 (3.016E-11)	-97.695 to -62.549	342.661
Extra-cellular water (Inverse)	Serial-Individual	Calculated	ECWi*s*	-3.569 (0.001)	-0.726 to -0.201	0.036	-3.416 (0.002)	-89.326 to -22.852	344.171
Total body water (Inverse)	Serial-Individual	Calculated	TBWi*s*	-2.111 (0.041)	-0.277 to -0.006	-4.685	-1.851 (0.072)	-35.008 to 1.569	350.64
Resistance	Parallel	Calculated	R_p	-2.601 (0.013)	-0.232 to -0.029	-0.086	-2.299 (0.027)	-29.842 to -1.893	350.154
Reactance	Parallel	Calculated	Xc_p	NS			NS		
Total body water	Parallel-Empirical	Calculated	TBW*p*	3.530 (0.001)	0.012 to 0.046	-3.295	3.865 (4.21E-4)	1.572 to 5.031	345.457
Intra-cellular water (Inverse)	Parallel-Empirical	Calculated	ICW*p*	4.223 (1.45E-4)	0.050 to 0.142	-9.89	**4.671 (3.70E-5)**	**6.460 to 16.344**	**336.237**
Extra-cellular water	Parallel-Empirical	Calculated	ECW*p*	-3.741 (0.001)	-0.036 to -0.011	-4.192	-4.193 (1.520E-4)	-4.012 to -1.399	343.855
Intra-cellular water	Parallel	Calculated	ICW*p2*	NS			NS		
Intra-cellular water	Parallel-Individual	Calculated	$ICWi_p$	-2.149 (0.038)	-0.388 to -0.012	-2.896	-2.333 (0.025)	-45.873 to -3.252	345.527
Extra-cellular water (Inverse)	Parallel-Individual	Calculated	$ECWi_p$	**-5.667 (2.00E-6)**	**-4.291 to -2.032**	**-8.894**	-6.102 (4.123E-7)	-523.851 to -262.859	339.182
Total body water (Inverse)	Parallel-Individual	Calculated	$TBWi_p$	NS			NS		
Total body water (Inverse)	Serial-Individual	Calculated	TBWi*s2*	-3.232 (0.003)	-0.593 to -0.136	-2.299	-2.678 (0.011)	-76.040 to -10.568	348.037
Total body water	Serial-Empirical	Calculated	TBW*s2*	3.623 (0.001)	0.013 to 0.045	-3.863	3.995 (2.87E-4)	1.610 to 4.919	344.662
Total body water (Inverse)	Serial-Individual	Calculated	TBWi*s3*	-4.578 (4.90E-5)	-0.559 to -0.216	-4.566	-4.537 (5.60E-5)	-68.908 to -26.388	343.872
Total body water (Inverse)	Parallel-Individual	Calculated	TBWi*p2*	-2.600 (0.013)	-0.313 to -0.039	-3.047	-2.790 (0.008)	-37.481 to -5.961	345.013
Total body water (Inverse)	Serial-Individual	Calculated	TBWi*s4*	-6.431 (1.462E-7)	-0.106 to -0.055	-1.32	-7.451 (6.079E-9)	-12.672 to -7.257	347.153
Body Weight		Measured					**5.961 (6.448E-7)**	**71.993 to 146.046**	**329.944**
Body Width (average)		Measured		**6.874 (3.644E-8)**	**0.023 to 0.042**	**-36.399**	4.252 (1.330E-4)	1.894 to 5.336	353.369
Body Length		Measured		7.778 (2.234E-9)	0.005 to 0.008	-31.792			

Table 5S. Predicting main effects of whole-body BIA measures on body water/moisture, proteins, ash/minerals in percent to body weight, and well-being (K). See explanation of abbreviations and marks in legend to Table 1S.

Electrical measures				Moisture			Proteins			Ash			K		
Name	Schema	Obtained	Symbol	t(p)	95% CI	AIC	t(p)	95% CI	AIC	t(p)	95% CI	AIC	t(p)	95% CI	AIC
Conductance module	Parallel	Measured [1]	*Y*	NS			NS			NS			NS		
Phase angle	Parallel	Measured [1]	ϕ	NS			-4.169 (1.70E-4)	-0.096 to -0.033	122.407	-2.422 (0.020)	-0.019 to -0.002	-3.418	2.489 (0.017)	0.003 to 0.033	53.569
Resistance	Serial	Calculated	*R*	NS			NS			NS			NS		
Reactance	Serial	Calculated	*Xc*	NS			-2.955 (0.005)	-2.165 to -0.405	116.746	-3.118 (0.003)	-0.288 to -0.061	-8.626	**3.828 (4.69E-4)**	**0.218 to 0.708**	**46.875**
Total body water	Serial-Empirical	Calculated	TBW*s*	1.982 (0.055)	-0.004 to 0.400	214.956	NS			1.744 (0.089)	-0.001 to 0.020	-2.796	NS		
Intra-cellular water (Inverse)	Serial-Empirical	Calculated	ICW*s*	2.104 (0.042)	-0.002 to -0.103	217.928	NS			2.260 (0.030)	3.46E-4 to 0.006	-0.831	NS		
Extra-cellular water	Serial-Empirical	Calculated	ECW*s*	-1.991 (0.054)	-0.128 to 0.001	217.590	NS			-2.379 (0.022)	-0.009 to -0.001	-1.538	NS		
Intra-cellular water	Serial	Calculated	ICW*s2*	NS			-4.531 (5.70E-5)	-4.387 to -1.678	114.392	-2.603 (0.013)	-0.866 to -0.108	-11.27	3.049 (0.004)	0.311 to 1.539	45.523
Intra-cellular water	Serial-Individual	Calculated	ICWi*s*	NS			-4.605 (4.50E-5)	-3.156 to -1.228	115.458	-2.780 (0.008)	-0.513 to -0.081	-9.714	5.297 (5.00E-6)	0.535 to 1.198	45.172
Extra-cellular water (Inverse)	Serial-Individual	Calculated	ECWi*s*	NS			NS			-2.039 (0.048)	-0.352 to -0.001	-8.625	1.958 (0.058)	-0.016 to 0.985	46.927
Total body water (Inverse)	Serial-Individual	Calculated	TBWi*s*	NS			NS			-1.933 (0.061)	-0.143 to 0.003	-6.870	NS		
Resistance	Parallel	Calculated	R_p	NS			NS			-2.660 (0.011)	-0.118 to -0.016	-6.703	2.223 (0.032)	0.016 to 0.335	49.058
Reactance	Parallel	Calculated	Xc_p	NS			NS			NS			NS		
Total body water	Parallel-Empirical	Calculated	TBW*p*	**2.217 (0.033)**	**0.020 to 0.441**	**214.518**	NS			2.060 (0.046)	1.99E-4 to 0.023	-3.346	NS		
Intra-cellular water (Inverse)	Parallel-Empirical	Calculated	ICW*p*	NS			NS			NS			NS		
Extra-cellular water	Parallel-Empirical	Calculated	ECW*p*	-2.073 (0.045)	-0.342 to -0.004	215.079	NS			-1.814 (0.078)	-0.017 to 0.001	-2.537	NS		
Intra-cellular water	Parallel	Calculated	ICW*p2*	NS			1.867 (0.070)	-0.030 to 0.741	120.049	NS			NS		
Intra-cellular water	Parallel-Individual	Calculated	$ICWi_p$	NS			NS			NS			NS		
Extra-cellular water (Inverse)	Parallel-Individual	Calculated	$ECWi_p$	NS			**-2.449 (0.019)**	**-16.791 to -1.593**	**112.987**	**-2.917 (0.006)**	**-2.261 to -0.408**	**-12.68**	3.639 (0.001)	1.720 to 6.033	42.415
Total body water (Inverse)	Parallel-Individual	Calculated	$TBWi_p$	NS			NS			NS			NS		
Total body water (Inverse)	Serial-Individual	Calculated	TBWi*s2*	NS			-2.173 (0.036)	-2.769 to -0.098	116.778	-3.321 (0.002)	-0.324 to -0.079	-8.888	**2.903 (0.006)**	**0.155 to 0.869**	**46.847**
Total body water	Serial-Empirical	Calculated	TBW*s2*	2.119 (0.041)	0.010 to 0.421	214.700	NS			1.915 (0.063)	-0.001 to 0.021	-3.092	NS		
Total body water (Inverse)	Serial-Individual	Calculated	TBWi*s3*	NS			-1.739 (0.090)	-2.196 to 0.167	117.585	-2.593 (0.013)	-0.279 to -0.034	-8.379	2.684 (0.011)	0.109 to 0.777	46.942
Total body water (Inverse)	Parallel-Individual	Calculated	TBWi*p2*	NS			NS			NS			NS		
Total body water (Inverse)	Serial-Individual	Calculated	TBWi*s4*	NS			-3.486 (0.001)	-0.410 to -0.109	119.936	-2.969 (0.005)	-0.061 to -0.012	-5.505	4.624 (4.30E-5)	0.058 to 0.150	49.583
Body Weight		Measured		NS			NS			1.838 (0.074)	-0.028 to 0.575	-10.94	NS		
Body Width (average)		Measured		NS			NS			1.763 (0.086)	-0.002 to 0.026	-5.724	NS		
Body Length		Measured		1.852 (0.072)	-0.004 to 0.087	216.029	NS			2.311 (0.026)	2.880E-4 to 0.004	-2.231	**-5.371 (4.00E-6)**	**-0.013 to -0.006**	**36.686**
